# Committed to climate action? Opportunities for scientific societies to lead the change we need in the world

**DOI:** 10.1186/s12966-026-01895-z

**Published:** 2026-03-30

**Authors:** Shilpa Dogra, Rodrigo Reis, Erica Hinckson, Ty Ferguson, Ester Cerin, Julia A. Wolfson, Sebastien Chastin, Delfien Van Dyck, Palma Chillón, Karim Abu-Omar, António L. Palmeira, ISBNPA Climate Action Committee

**Affiliations:** 1 Faculty of Health Sciences (Kinesiology), Ontario Tech University, 2000 Simcoe St N, Oshawa, ON L1G-0C5 Canada; 2 People, Health and Place Unit, Prevention Research Center, School of Public Health, Washington University in St. Louis, One Brookings Drive, St. Louis, MO USA; 3 Human Potential Centre, School of Sport, Exercise and Health, Faculty of Health and Environmental Sciences, Auckland University of Technology, Auckland, New Zealand; 4 Alliance for Research in Exercise, Nutrition, and Activity (ARENA), University of South Australia, Adelaide, South Australia Australia; 5 Mary MacKillop Institute for Health Research, Australian Catholic University, Melbourne, VIC Australia; 6 School of Public Health, The University of Hong Kong, Hong Kong, Hong Kong SAR, China; 7 Department of International Health, Johns Hopkins Bloomberg School of Public Health, Baltimore, MD USA; 8 Department of Health Policy and Management, Johns Hopkins Bloomberg School of Public Health, Baltimore, MD USA; 9 School of Health and Life Sciences, Glasgow Caledonian University, Glasgow, Scotland, UK; 10 Department of Movement and Sports Sciences, Ghent University, Ghent, Belgium; 11 Department of Physical Education and Sports, Faculty of Sport Sciences, Sport and Health University Research Institute (IMUDS), University of Granada, Granada, Spain; 12 Department of Sport Science and Sport, Friedrich-Alexander Universität Erlangen-Nürnberg, Erlangen, Germany; 13 CIDEFES, Universidade Lusófona and CIFI2D Universidade Do Porto, Lisbon and Porto, Porto, Portugal; 14 International Society of Behavioral Nutrition and Physical Activity, Baltimore, USA

**Keywords:** Climate change, Sustainability, Conferences, Behaviour change, Research

## Abstract

The climate crisis is undoubtedly compromising human and planetary health. As behaviour change scientists, we must reflect on the behaviours we are promoting, as well as the research and professional practices we engage in, to ensure we are prioritizing human and planetary health. This commentary focuses on issues within the scope of scientific/academic societies by sharing our journey as the Climate Action Committee for the International Society for Behavioural Nutrition and Physical Activity. First, we provide details on the structure and function of our committee, then we provide insights into the GHG emissions associated with running our society, and finally, we report on our journey thus far, sharing the strategies we have identified that align with current evidence on climate change. We also address the tensions associated with climate action within scientific societies, and hope to begin a dialogue with others to come up with creative solutions that continue to support research and researchers, while addressing the urgency of our climate crisis.

**Supplementary Information:**

The online version contains supplementary material available at 10.1186/s12966-026-01895-z.

## Context

The International Society of Behavioral Nutrition and Physical Activity (ISBNPA), established in 2000, is a scientific society dedicated to stimulating and promoting innovative and impactful research in behavioral nutrition and physical activity, to enhance human and planetary health worldwide. Our society is a tightly knitted, friendly, welcoming and thriving scientific community centred around our Annual Conference, which brings together between 800–1,000 researchers, and our Journal (International Journal of Behavioral Nutrition and Physical Activity), which publishes high quality, open access research.

In 2022, ISBNPA developed a new strategic plan centered on four main goals: 1) Climate Action, 2) Research Impact, 3) Inclusive Culture, and 4) Growing the Next Generation. A Climate Action Committee was established in 2022 as part of this commitment. Their first action in 2023 led to an update of ISBNPA's mission. The mission, which previously focused on human health, was updated to include the term "planetary health", indicating a deep commitment to climate action and the interconnected nature of human and planetary health.

Although ISBNPA has been taking climate-related actions since 2015, the establishment of a formal committee has empowered ISBNPA to bring climate issues to the forefront within its organizational structure. The objectives of this committee are to: 1) find strategies to support ISBNPA with reducing its carbon footprint, 2) engage the ISBNPA research community in climate change issues, and 3) champion positive contributions to behavior change science, related to physical activity and nutrition, in addressing climate change. The annual budget for this committee is $10,000 USD per year. The establishment of this committee has allowed ISBNPA to develop and implement a comprehensive action plan.

In this commentary, we share our experiences, ideas, actions, and failures with the hope that they will encourage and support other scientific societies to engage in meaningful and effective actions that match the urgency and scale of climate change.

### The climate cost of a scientific society

There is unequivocal evidence that climate change is a result of human activity, and that all economic activities significantly contribute to GHG emissions. Researchers, scientific societies, and institutions worldwide are striving to reduce their carbon footprint while continuing to support the advancement of science and knowledge. Although many scientific institutions have committed to Net Zero by 2050, taking meaningful strides toward this goal has been challenging [1]. Similarly, while ISBNPA has pledged to half its GHG emissions by 2030, it does not appear we will meet our targets due to tensions within the structural organization focused on competing priorities. We are at a critical point, and the research enterprise is facing an ethical dilemma related to climate change. Using the concept of H. Shoe (1993), those involved with research at all levels need to question whether the GHG emissions produced by their work and activities are worthwhile [2]. For example, should traveling to conferences be considered “luxury emissions”, that is, emissions that should be cut first, or are they “subsistence emissions”, that is, those that are essential for survival and cannot be cut.

ISBNPA’s main activity as a society is the annual conference. Travelling to such conferences is an important part of scientific life, as it enables collaboration and provides necessary exposure to peers. It is also a requirement of a scientific career, as presentations at conferences are expected for promotions, funding, and employment. While some researchers, particularly those with established careers from higher-income countries, have discontinued travelling for conferences to reduce their emissions, it is important to recognize that for others, conferences are not a luxury emission, but are a subsistence emission essential for their career. As such, structural changes are required to reduce emissions related to conference travel.

The COVID-19 pandemic temporarily reduced emissions related to scientific conference participation due to travel restrictions, providing us with opportunities to innovate and learn [3]. For example, by switching annual in-person conferences to e-meetings (virtual environment), the 2021 Consortium of Universities for Global Health (~ 2,000 registrants) found a carbon footprint reduction of 2,436 metric tons of CO_2_, equivalent to 2,994 acres of deforestation, in one year [4]. There are other ways to reduce conference-related emissions, but solely focusing on conferences may be insufficient. According to an analysis of emissions related to a PhD, conferences only contribute to approximately a third of GHG emissions related to research [5]. For example, in laboratory-based research, single-use plastics and cold storage (freezers) can also significantly contribute to GHG emissions [6]. Moreover, electronics and artificial intelligence used by all researchers across the globe contribute to GHG emissions [7]. While all researchers use technology, a growing number of behaviour change researchers are relying on data collection methods that use carbon intensive equipment or interventions (e.g. smart-phone applications and trackers), and generate big data that requires advanced data management and analytic techniques (e.g. online software and platforms). Therefore, the research community, including scientific societies, must explore innovative solutions to mitigate GHG emissions directly related to their work.

As behavioral nutrition and physical activity researchers, there are also indirect emissions related to the promotion of certain behaviors that need to be considered. For example, should we promote physical activity for health reasons if this involves driving a car to a nature park to go for a hike? Or eating a heart healthy diet that is primarily based on foods grown in distant parts of the world? In the field of physical activity, the inter-relationship between physical activity and climate change has been discussed and documented [8–11]. Mitigation strategies primarily focus on reducing emissions by promoting activities that benefit both the health of the planet and its inhabitants, such as active travel. Adaptation strategies should focus on protecting the most vulnerable communities from the acute (e.g. wildfire smoke) and long-term/gradual effects (e.g. rising sea levels and excessive heat) of climate change, thereby ensuring that we do not exacerbate physical activity inequities [11]. In the field of nutrition, there are numerous opportunities to support behavior change related to healthy eating, access to healthy food in urban environments, and proper nutrition throughout the lifespan. [9]. For example, a planetary health diet (with reduced red meat intake) is advocated for the health of both humans and the planet [12], while a review of 16 West African countries identified several challenges that require evidence-informed insights, highlighting areas for immediate and targeted research in the area of nutrition and sustainability [13]. Our scientific society and others have an important role to play in promoting and supporting research that contributes to impactful climate action within their respective fields.

### Our actions and identified opportunities

#### Annual conference

We have implemented several measures to reduce GHG emissions associated with ISBNPA’s annual meeting (Table 1).

**Table 1:** ISBNPA’s strategies, actions, and identified opportunities to reduce GHG emissions related to the annual conference

Strategies	Actions	Opportunities
Frequency of the Conference	2023, A recommendation was made to the Executive Committee that the conference be changed from an annual to a biennial conference. The rationale was that travel represents > 90% of the society's GHG emissions, and thus this was essential to meet the target to reduce emissions by 50% by 2030. The Executive Committee turned this down citing concerns related to impacts on early career-researchers, and loss of membership and funding	To overcome the concerns listed by the Executive Committee, we are exploring the possibility of developing local chapters to provide biannual in-person activities, and synchronizing the timing of conferences with other related scientific societies
Location	2019, Requirement that bids to host the conference include an assessment of the climate impact of the annual meeting is enacted 2022, The annual conference was held in a hybrid format, with the cost mirroring those of an in-person event. Online participants felt excluded from in-person discussions and activities 2024, Mapping approach to identify geographic areas with greater proximity to existing members, hence lowering GHG emissions (See Supplementary Fig. 1) 2024, Commitment to rotation of the annual meetings, with the European region as hosting continent on alternate years, projecting 25% reduction of GHG emissions over a 4-year period	Foster local chapter events to reduce travel to annual conferences Prioritize locations that are welcoming to colleagues from low-income countries
Member Engagement	2022, Survey on climate action activities was included in the conference registration (e.g., research areas intersecting with climate change, travel carbon footprint) 2023, Climate Action keynote speaker at the conference in Sweden (joined online) 2024, Climate Action Committee panel at the conference, with experts at the intersection of climate action, equity, and physical activity and nutrition research 2025, Climate Action annual report was presented at the conference, and a Climate Action Committee booth was set-up to raise awareness	Incentives to travel by sustainable modes (e.g. lower conference registration if the individual travelled by train, although this needs to be balanced with equity related priorities) Require a GHG budget be included in abstracts to raise awareness about the carbon footprint of research Provide education around GHG data/numbers so that researchers can understand their personal and professional carbon footprint (e.g. gamified opportunities at the conference)
Food	2017, Annual meeting bids were required to consider food sustainability in their bids 2023, Food offered at the annual conference was plant-based, from local producers, and seasonal 2024, Food offered was both plant-based and buffet style to reduce food waste	Requiring seasonal and local vegetarian meals that emphasize planetary diet and farm-to-table types of sustainable practices
Others	2023, Emissions calculator added to the Annual Meeting Website with sustainability tips and strategies for lowering participants GHG emissions	Require keynote speakers to be regional or local, or to join online Require all keynotes to address opportunities related to climate change Create an award category for research on planetary health Create opportunities to discuss and share ways in which members can integrate climate action focused work into their own research programs

#### Carbon footprint monitoring

**Fig. 1:**
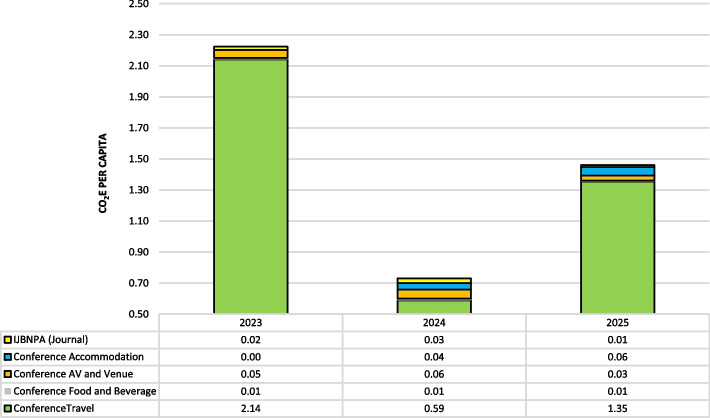
Per Capita Emissions

In 2022, we invested efforts and funds in training some of our staff in carbon accounting to put a regular monitoring system in place. In 2023, we published our first GHG emissions report, with assistance from external advisors. Figure 1 provides the 2023–2025 ISBNPA GHG emissions. This chart clearly demonstrates that the biggest contributor to ISBNPA’s GHG emissions is the annual conference.

In the 2024 report, our overall GHG was reduced by 50%, both in absolute and per capita values. This was primarily because fewer people attended the conference (particularly international attendance was markedly reduced), and because the hosting city was close to the primary location of many of our members (i.e., the 2024 annual meeting was held in Omaha, Nebraska and was predominantly attended by colleagues from the US). Our preliminary calculations for 2025 showed a steep increase in GHG emissions due to the conference's location in Auckland, New Zealand, geographically distant from the majority of our membership. The conference was originally scheduled in 2020 but was then postponed due to COVID-19 to 2025; we decided to keep it knowing that it would have this impact on our emissions.

We developed a comprehensive Carbon Footprint Management Plan by the Greenhouse Gas Protocol Standards and Guidance, incorporating emissions calculators tailored to ISBNPA activities (e.g., costs associated with this journal). The external advisors validated and reviewed all methods available in our ISBNPA 2024 Carbon Footprint Management Plan. Since 2023, ISBNPA has included a Green Box in its newsletters, offering practical tips on how to reduce research-related greenhouse gas (GHG) emissions.

#### Research related

The Climate Action Committee is engaging members in a mapping activity to identify collaborative opportunities on climate change and physical activity. We plan to lead a similar activity on the nutrition side. The goals of this activity are to: a) identify experts who can support the society in leading additional initiatives, and b) support researchers with considering climate change and climate action in their research agenda. Ideas and examples exist for the latter [14]; however, it is critical that we undertake this exercise within the society to ensure that behavior change researchers are engaged with the ideas generated. There are immediate and clear opportunities for climate change research, leveraging ISBNPA’s expertise. As such, current opportunities being explored to encourage research on climate change include providing seed funding for research focused on planetary health, offering early-career awards to those working on climate-related initiatives, establishing a new Special Interest Group at ISBNPA, and leveraging special issues in the journal to catalyze new research on behavior change and climate change. As a society of behavior change researchers, we have a meaningful role to play in supporting people with making sustainable changes to their movement and dietary patterns by developing and implementing evidence-informed interventions that lead to long-term behavior change.

#### Alignment with other strategic priorities

Having a Climate Action Committee and a structure that requires the engagement of our Executive Committee ensures that other strategic priorities, such as equity, diversity, and inclusion, particularly as it relates to low-income countries, and support for early-career researchers are not compromised. Some opportunities align with all strategic priorities that support the Climate Action Committee in reducing the GHG emissions of ISBNPA. One such *triple win* type of opportunity that has not yet been mentioned is the value of *frugality,* that is, sparing use of resources to reduce consumption and waste. For example, we have reduced costs related to printing conference agendas, providing free swag, and using water bottles at the annual conference, and thus over the long term, we may be able to lower the carbon footprint as well as the cost of the conference, making it more accessible to early-career researchers and researchers from low-income countries. Frugality is a value that can be applied more broadly to the research enterprise as well. For example, many researchers have office spaces on campus that are not used regularly, but are heated and cooled all year round, highlighting the need for researchers to give up luxuries once considered essential. The cost savings could be re-invested into early career researchers or those from low to middle income countries. Furthermore, by supporting climate change-related research among early-career researchers or those from equity-deserving groups, we can contribute to the creation of new knowledge that has a positive impact on planetary health.

### Moving forward

Climate action must be balanced with efforts to ensure scientific societies remain open and inclusive to researchers worldwide. Balancing different priorities does not mean compromising the quality or integrity of our actions, the life of the society, or the career trajectories of our members. Clearly, the society must do more, and must do more dramatically and aggressively, to reduce its carbon footprint. Yet, this will only be possible if we can provide alternatives to support our members and ensure we remain financially viable.

For now, the society has taken an informed conservative view. For example, as a scientific society, we recognize that our membership is primarily composed of individuals from high-income countries, and the costs of membership and annual conferences are often prohibitive for many colleagues in middle- and low-income countries. Thus, while we will focus on hosting our annual conference in countries where most of our membership resides, we will also take the annual conference to locations that allow other colleagues to participate. This balance ensures we can reduce our GHG emissions over four years, rather than concentrating on each year in isolation. We also hope that by being vocal and raising the issue of climate action across various platforms and functions, that we will engage early-career researchers, and our research community as a whole, in areas of inquiry that intersect with planetary health and climate change. There is an urgent and clear opportunity for ISBNPA researchers to apply their expertise in behavior and collective change to support climate action. This work must begin with increasing climate literacy and meaningful interdisciplinary cooperation with climate experts among our research community so we can make substantial advances in this space.

There also exist opportunities for the Climate Action Committee and members of ISBNPA to engage in meaningful and impactful advocacy, both within the society, and with external partners. For example, the Climate Action Committee will consider approaches taken by others such as those in Health Psychology and Behavioral Medicine who recommend engaging policy makers and business leaders, the public, and their own members through advocacy and communities of practice [15]. Engagement with external partners is ongoing. We regularly collaborate with other academic societies to support one another with climate action. There is also an opportunity to join groups such as Global Climate and Health Alliance and the Planetary Health Alliance to further strengthen work in this space.

## Conclusion

As scientific societies, we must create an environment that can foster changes that meet the urgency of our current climate crisis, while respecting the rigor and value of scientific inquiry. We encourage all researchers and scientific societies to reflect on their contributions to climate action and to take immediate steps to respond to the climate crisis. We hope that this commentary encourages an open dialogue between scientific societies, so that we can innovate and creatively address the many ways we can advance the climate agenda in our research enterprises.

## Supplementary Information

Supplementary Material 1: Supplementary Figure 1. Screenshots of the Mapping Tool (mapbox) - identifying geographic areas with greater proximity to the primary location of our members.

## Data Availability

No datasets were generated or analysed during the current study.

## Abbreviations

GHG: Green House Gases
ISBNPA: International Society for Behavioral Nutrition and Physical Activity
CO_2_: Carbon Dioxide
USD: United States Dollar
