# Supplementary figures and images for: Committed to climate action? Opportunities for scientific societies to lead the change we need in the world
Source: Int J Behav Nutr Phys Act. 2026 Mar 30;23:30. doi: 10.1186/s12966-026-01895-z (PMC13034594; doi:10.1186/s12966-026-01895-z)

## Slide 1
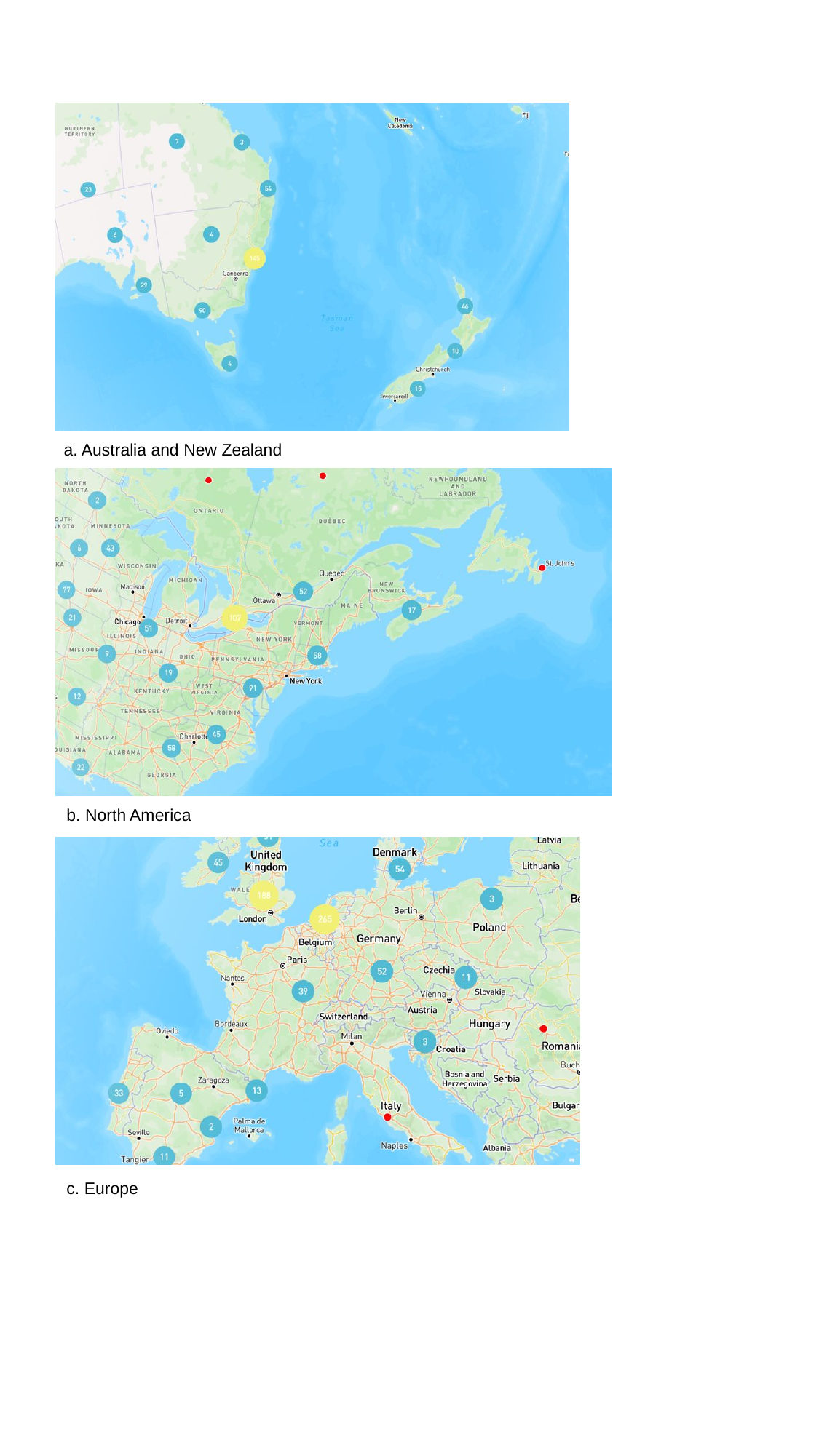

a. Australia and New Zealand
b. North America
c. Europe

Supplement: Supplementary file 1 — Supplementary Material 1: Supplementary Figure 1. Screenshots of the Mapping Tool (mapbox) - identifying geographic areas with greater proximity to the primary location of our members. [file 12966_2026_1895_MOESM1_ESM.pptx]
